# A Guide to the Alterations in the British Pharmacopœia (1885)

**Published:** 1889-12

**Authors:** 


*A Guide to the Alterations in the British Pharmacopœia* (1885). By PROSSER JAMES, M.D. Third Edition. London: J. & A. CHURCHILL. 1889.

This is our first acquaintance with this book, which is a methodical review of the changes made in our most recent Pharmacopœia. The criticisms are fearless, and are made with much shrewdness, and often with humour. It is a book to have.

